# The Crucial Role of Divalent Metal Ions in the DNA-Acting Efficacy and Inhibition of the Transcription of Dimeric Chromomycin A3

**DOI:** 10.1371/journal.pone.0043792

**Published:** 2012-09-12

**Authors:** Chun-Wei Hsu, Show-Mei Chuang, Wen-Ling Wu, Ming-Hon Hou

**Affiliations:** 1 Institute of Genomics and Bioinformatics, National Chung Hsing University, Taichung, Taiwan; 2 Institute of Biomedical Sciences, National Chung Hsing University, Taichung, Taiwan; 3 Biotechnology Center, National Chung Hsing University, Taichung, Taiwan; 4 Department of Life Science, National Chung Hsing University, Taichung, Taiwan; Aligarh Muslim University, India

## Abstract

Chromomycin A3 (Chro) is capable of forming a stable dimeric complex via chelation with Ni(II), Fe(II) and Co(II). According to the circular dichroism study, the dimer conformations are significantly different among the Fe(II)-, Co(II)-, and Ni(II)-containing dimeric Chro complexes; however, the dimer conformations were preserved at high temperatures. Furthermore, we conducted a systematic study to determine the effects of these divalent metal ions on the DNA-acting efficacy of dimeric Chro, including its DNA-binding affinity, DNA stabilization capacity, DNA cleavage activity, and the inhibition of transcription both *in vitro* and within cells. Kinetic analyses using surface plasmon resonance (SPR) showed that Ni^II^(Chro)_2_ exhibited the highest *K*_a_ with a value of 1.26×10^7^ M^−1^, which is approximately 1.6- and 3.7-fold higher than the *K*_a_ values obtained for Co^II^(Chro)_2_ and Fe^II^(Chro)_2_, respectively. The *T_m_* and Δ*G* values for the DNA duplex increased after the addition of drug complexes in the following order: Ni^II^(Chro)_2_>Co^II^(Chro)_2_>Fe^II^(Chro)_2_. In the DNA integrity assays, the DNA cleavage rate of Co^II^(Chro)_2_ (1.2×10^−3^ s^−1^) is higher than those of Fe^II^(Chro)_2_ and Ni^II^(Chro)_2_, which were calculated to be 1×10^−4^ and 3.1×10^−4^ s^−1^, respectively. Consistent with the SPR and UV melting results, Ni^II^(Chro)_2_ possesses the highest inhibitory effect on *in vitro* transcription and c-myc transcription within cells compared to Co^II^(Chro)_2_ and Fe^II^(Chro)_2_. By comparing the cytotoxicity among Co^II^(Chro)_2_, Fe^II^(Chro)_2_, and Ni^II^(Chro)_2_ to several cancer cell lines, our studies concluded that Ni^II^(Chro)_2_ displayed more potential antitumor activities than Co^II^(Chro)_2_ and Fe^II^(Chro)_2_ did due to its higher DNA-acting efficacy. Changes to the divalent metal ions in the dimeric Chro complexes have been correlated with improved anticancer profiles. The availability of new metal derivatives of Chro may introduce new possibilities for exploiting the unique properties of this class of compounds for therapeutic applications.

## Introduction

**Figure 1:**
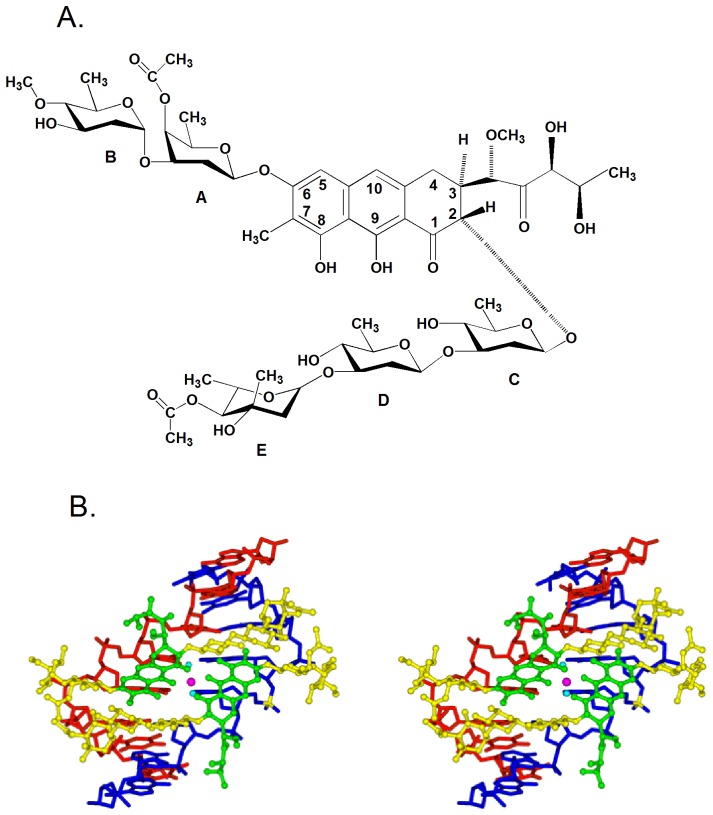
The binding of chromomycin A3 to DNA. (**A**) Chemical structure of chromomycin A_3_ (Chro). (**B**) Stereoscopic drawings of the Mg(II)-coordinated Chro-(TTGGCCAA)_2_ complex viewed from the minor groove direction (Chro is depicted in a ball-and-stick representation, and the DNA is represented by the skeletal line drawing). (PDB:1VAQ).

Chromomycin A_3_ (Chro), a glycosidic metalloantibiotic, is a member of the aureolic family of drugs isolated from *Streptomyces griseus* [1]–[3]. This drug contains di- and trisaccharide moieties connected to a β-ketophenol chromophore via O-glycosidic bonds arranged in a 2,6 relationship around the anthracene ring, with the disaccharide at the 2 position and the trisaccharide attached at the 6 position (**Figure 1A**) [4]. Chro has been used for the treatment of malignant diseases, such as testicular tumors, and is of limited use in the treatment of hypercalcemia [2], [5]. The antitumor properties of Chro can be attributed to its inhibitory effects on transcription during cell proliferation [6]. Chro has also demonstrated the capacity to downregulate the expression of many cancer-related genes that bear GC-rich motifs in their promoters, such as the c-myc proto-oncogene that regulates cell proliferation [4], [7]. Furthermore, Chro is a potent inhibitor of the neuronal apoptosis induced by oxidative stress, indicating that Chro could potentially play a role in neurological therapy [8]. In addition, Bianchi *et al*. demonstrated that Chro is a powerful inducer of erythroid differentiation of K562 cells by binding to the human γ-globin promoter and altering the pattern of protein binding to this promoter [9].

Metal ions play an indispensable role in the actions of some synthetic and natural metalloantibiotics, and they are involved in specific interactions of these antibiotics with biomolecules [10]. Previous optical spectroscopic and structural studies showed that divalent metal ions, such as Mg(II), Zn(II), or Co(II), are necessary for Chro to bind to DNA duplexes at GC-rich sequences that are at least three base pairs long [11]–[13]. The solution structures of Chro bound to a DNA duplex were analyzed using NMR spectroscopy, and the crystal structure of Chro-DNA complex has already been reported. This structure reveals that the metal-coordinated dimer of Chro causes a kink in the DNA on the minor groove side and induces a local duplex conformational change [14]–[19] (**Figure 1B**). The divalent metal ion in the complex has an octahedral coordination with two water molecules acting as the fifth and sixth ligands (**Figure S1**). Previous studies have reported that the binding of Chro to DNA can be achieved only using divalent metal ions whose ionic radii are less than 85 pm [20]. The interactions between drugs of the aureolic family and divalent metal ions have been evaluated for years, but the alteration of the metal ions that amplify the potential of these drugs in disease therapy seems to vary [4], [13], [21].

In this study, we found that Chro is capable of forming a dimeric complex via chelation with Fe(II), Ni(II) and Co(II) in the absence of a DNA duplex. According to the circular dchroism (CD) study, although the dimer conformations are significantly different among the Fe(II)-, Co(II)-, and Ni(II)-containing dimeric Chro complexes in the absence of DNA binding, the dimer conformations were predominately preserved at high temperatures. Furthermore, we conducted a systematic study to determine the effects of these divalent metal ions on the DNA-acting efficacy of dimeric Chro, including its DNA-binding affinity, DNA stabilization capacity, DNA cleavage activity, and the inhibition of transcription both *in vitro* and within cells. Finally, the cytotoxicities of Fe(II)-, Co(II)-, and Ni(II)-containing dimeric Chro complexes in several cancer cell lines were also evaluated and compared in this study. Changes to the divalent metal ions in the dimeric Chro complexes have been correlated with improved anticancer profiles. The availability of new metal derivatives of Chro may introduce new possibilities for exploiting the unique properties of this class of compounds for therapeutic applications.

## Materials and Methods

Synthetic DNA oligonucleotides were purified using gel electrophoresis. Chro was purchased from Sigma Chemical Co. (St. Louis, MO). The absorbance measurements were obtained using a quartz cuvette in a Hitachi U-2000 spectrophotometer. The Chro concentrations were estimated using an extinction coefficient of 8,800 M^−1^ cm^−1^ at 405 nm [22]. The oligonucleotide concentrations were determined according to Beer's law (A = ε·b·c, where A is the optical density at 260 nm, ε is the extinction coefficient, b is the cell path length of 1 cm, and c is the DNA concentration in M). The extinction coefficient of the plasmid DNA is 6,600 M^−1^ cm^−1^. The oligomer extinction coefficients were calculated using tabulated values for the monomer and dimer extinction coefficients as a reasonable estimate [23].

### CD measurements

The hairpin DNA d-TTGGCCAATGTTTGGCCAA was used in the CD experiments. CD spectra were collected between 520 and 200 nm at a resolution of 1 nm using a JASCO-815 spectropolarimeter. The temperature was controlled using a circulating water bath. All spectra are represented as the average of three measurements. The methods used for the CD spectral analysis have been described previously [24], [25].

### DNA melting studies

The methods used for the DNA melting analysis have been described previously [26]. The UV absorbance vs. temperature profiles were measured using a JASCO V560 UV/VIS spectrophotometer by monitoring the sample absorption (in OD) at 260 nm. The sample cell was equipped with a Peltier-type cell holder (EHC-441), and the temperature was regulated by a programmer (JASCO TPU-436). The concentration of the duplex DNA in each sample was 3 µM in 20 mM Tris–HCl at pH 7.3 in 50 mM NaCl. The experiments were performed by increasing the temperature at a rate of 0.5°C/min from 0 to 100°C, and the temperature was recorded every 30 sec. The data set of each melting curve was normalized to minimize the variations in each experiment because *T*_m_ is independent of the DNA concentrations. To obtain van't Hoff transition enthalpies, the UV melting curves were evaluated so that the plot of the experimental absorbance vs. temperature could be converted into a curve of melted fractions vs. temperature. The plots of the melted fractions in single strands (*f*) vs. temperature (*T*) were calculated by fitting the melting profile to a two-state transition model [27]. The *T*_m_ values were evaluated directly from the temperature at *f* = 0.5.

The equilibrium constants and thermodynamic parameters of the DNA interacting with and without drug complexes were calculated using Thermal Melt Analysis System (Varian, Inc). Briefly, the equilibrium constant, *K*, at a given temperature, *T*, was calculated using the equation, *K* = (1−*f*)/[(C_T_/*n*)^(*n*−1)^ *f^n^*], where *f* is the melted fraction in single strands, *n* is the molecularity of the reaction, and *C*_T_ is the total concentration of strands. The enthalpy change, Δ*H*, was determined from the temperature dependence of the equilibrium constant, *K*. Δ*H* was calculated from the slope of a plot of ln *K*_a_ vs. 1/*T* according to the equation, ln *K*_a_ = −Δ*H*/*RT*+Δ*S*/*R*, where Δ*S*, which is the entropy change, was obtained from the ordinate at the origin of the fitted line. The free energy change, Δ*G*, at 25°C was calculated from the following relationship: Δ*G* = Δ*H*−*T*Δ*S*.

### DNA binding analysis

The affinity, association and dissociation between the drug and the DNA duplexes were measured using a BIAcore 3000 A surface plasmon resonance (SPR) instrument (Pharmacia, Uppsala, Sweden) with a SensorChip SA5 from Pharmacia that monitored changes in the refractive index at the sensor chip's surface. These changes are generally assumed to be proportional to the mass of the molecules bound to the chip and are recorded in resonance units (RU). The 5′-biotin-labeled hairpin DNA, biotin-d(TTGGCCAATGTTTGGCCAA), that were purified using PAGE were used in the SPR experiments (the hairpin loop is underlined). To control the amount of DNA bound to the SA chip surface, the biotinylated oligomer was manually immobilized onto the surface of a streptavidin chip. Solutions of the metal-derived Chro complexes buffered with 20 mM Tris–HCl at pH 7.3 in 50 mM NaCl were prepared and used. Different concentrations of the complexes were passed over the surface of the chip for 180 sec at a flow rate of 10 µL min^−1^ to reach equilibrium; one of the flow cells remained blank as a control. Blank buffer solution was then passed over the chip to initiate the dissociation reaction, and this procedure was continued for 300 sec to complete the reaction. The surface was then recovered by washing it with 10 µL of a 10 mM HCl solution. The binding constant was calculated as previously described [28]. The sensorgrams for the interactions between the hairpin DNA duplex and the drug were analyzed using version 3 of the BIA evaluation software.

### Measurement of DNA strand breakage using plasmid DNA

*E. coli* (DH-5α) were transformed with pTYB11 and grown in LB medium. The plasmid DNA was then purified using a Qiagen plasmid purification kit (Valencia, CA). Reagents were added in the following order: phosphate buffer (pH 7.3), drug complexes, supercoiled plasmid DNA, and H_2_O_2_. (Note that phosphate, not Tris, buffer was used in the analysis of DNA strand breakage because Tris has been previously shown to generate formaldehyde upon reaction with hydroxyl radicals) [29]. The samples were incubated at 37°C at various time points, and the reactions were stopped with thiourea prior to electrophoresis in a 1% agarose gel followed by ethidium bromide staining for analysis. Quantification of the DNA bands on the gel was achieved using the Uniphoto Band Tool software.

### RNA polymerase assays

During *in vitro* transcription, T7 RNA polymerase was used for the RNA polymerase assay (Promega). The linearized DNA template pTRI-β-actin-mouse and a T7 primer were used in this study. The primer-template complex in the presence or absence of dimeric Chro-metal complexes was heated to 95°C for 5 min and was then equilibrated for 10 min at 4°C in reaction buffer (40 mM Tris–HCl at pH 7.5, 7 mM MgCl_2_, 0.1 mM DTT). The enzyme and NTP reaction solution were micropipetted into the DNA-drug complex solution, which was then incubated at 37°C for 10 min. The final enzyme and dNTP concentrations were 0.5 U and 2 mM, respectively. An aliquot (10 µl) of the reaction buffer was removed and quenched by the addition of loading buffer (50% glycerol, 0.25% bromophenol blue, and 0.25% xylene cyanol), and the solution was heated to 80°C for 10 min. The reaction products were examined using denaturing gel electrophoresis at 100 V in 10% polyacrylamide (10 cm×10.5 cm×0.75 mm) in TBE buffer, with 12 M formamide as a denaturant. The gels were stained with SYBR Green, and the bands were detected in the gel using Imagemaster VDS (Pharmacia).

### Cell culture and cell viability assay

The human cervical carcinoma (HeLa) and human oral squamous carcinoma (HSC-3) cell lines (ATCC) were a gift from Dr. Alan Yueh-Luen Lee **(**National Health Research Institutes, Miaoli, Taiwan). The human alveolar epithelial carcinoma cell line (A549) was provided by the American Type Culture Collection (Rockville, MD). The cells were maintained in Dulbecco's modified Eagle medium (DMEM) supplemented with 10% fetal bovine serum (FBS) and 1% antibiotics. The cells were incubated at 37°C in a humidified atmosphere with 5% CO_2_. Cellular proliferations were evaluated using the colorimetric MTS assay (CellTiter). The MTS tetrazolium compound is bioreduced by living cells into a purple water-soluble formazan product in culture medium as a cell-viability indicator. For the cell proliferation assay, cells were seeded into 96-well plates at a density of 5×10^3^ cells/well and incubated with drugs for 24 h. After 24 h, the original medium was removed and replaced with the drug complexes in fresh medium. After an additional 24 h, the MTS solution was added, and the plates were incubated in a moist chamber at 37°C for an additional 1 h. The optical density was measured at 490 nm in an ELISA reader. At least three independent experiments were performed to obtain the results for a statistical analysis.

### Reverse transcription and real-time PCR

HeLa cells were seeded into 6-well culture plates at a density of 1×10^6^ cells and incubated for 24 h. After 24 h, the cells were treated with different drugs and then incubated for 6 h. Total RNA was isolated from HeLa cells using Trizol reagent. To remove residual traces of DNA, the RNA samples were treated with DNase I. The total RNA concentrations were determined from the absorbances at 260 nm. For cDNA synthesis, 1 µg of the total extracted RNA was reverse-transcribed using Improm-II reverse transcriptase and random primers (Promega) in a final volume of 20 µl. PCR amplification of cDNA was performed in a final volume of 20 µl containing 10× reaction buffer, 50 mM MgCl_2_, 10 mM dNTPs, 50 ng/µl primers for β-actin (forward primer: 5- AGCGGGAAATCGTGCGTGACA-3 and reverse primer: 5-GTGGACTTGGGAGAGGACTGG-3) and c-myc (forward primer: 5-AAGGCTCTCCTCTGCTTAG-3 and reverse primer: 5-CTCTCCTCGTCGCAGTAGAAATAC-3), 2 U of Taq DNA polymerase, and 2 µl of cDNA. The PCR products were subjected to gel electrophoresis on 2% agarose gels in Tris-acetate-EDTA (TAE) buffer at 100 V for 40 min. After staining with ethidium bromide for 15 min, the gels were destained in water for 1 h and were visualized and photographed under UV light using a UV-Photo Imager (EZlab). The intensities of the DNA bands and the blank bands were determined using BIO-RAD Quantity One software.

### Transfection and luciferase reporter assays

HeLa cells were seeded into 6-well plates at a density of 1×10^6^ cells/ml for 24 h before transfection. The c-myc promoter luciferase reporter vector was purchased from Addgene, Inc. (Cambridge, MA). A 1-µg sample of the plasmid was transfected into HeLa cells using jetPEI™ according to the manufacturer's protocol. After 6 h, the medium was replaced with fresh medium containing the desired concentrations of each compound, and the cells were then incubated for an additional 18 h. After treatment with the drugs, the medium was removed, and the cells were gently rinsed with 1× PBS. The cells were lysed with Glo lysis buffer with continuous pipetting at 4°C for 10 min. After cell lysis, the samples were centrifuged at 10,000 rpm for 5 min, and cell extracts were then prepared to determine protein concentrations. Luciferase assays were performed using the luciferase assay system (Promega). The luciferase activities were normalized to total protein concentrations. Each measurement was obtained in triplicate.

### Statistical analysis

The cell assay results are expressed as the standard errors of the mean (S.E.M.), S_X_ = S/n^1/2^, where S is the standard deviation and n is the number of experiments. The mean values were compared using a two-way analysis of variance (ANOVA) (SigmaStat v.2, SPSS Inc., Chicago, IL). The level set for statistical significance was *p*<0.05.

## Results

### Effect of divalent metal ions on the dimer formation of Chro

**Figure 2:**
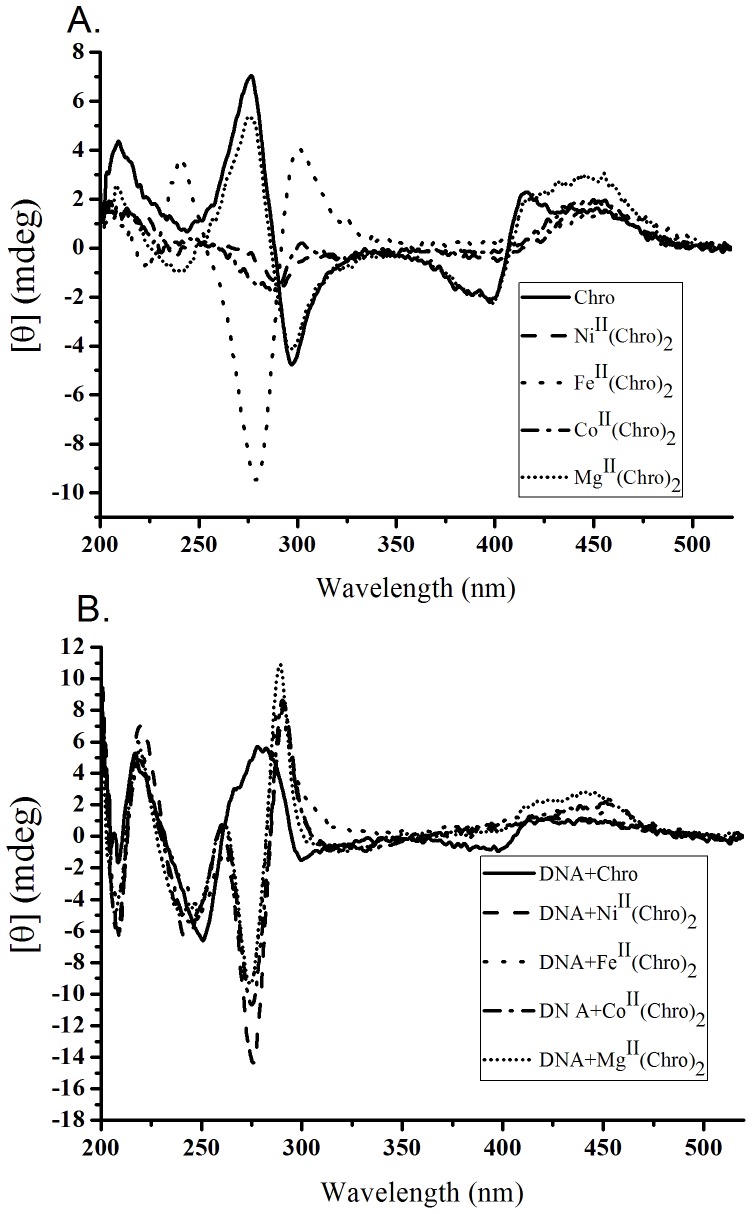
The conformational changes of Chro upon binding to divalent metal ions. (**A**) CD spectra of Chro in the presence of various divalent metal ions. The drug concentration was 0.04 mM in a 20 mM Tris-HCl buffer (pH 7.3) at 20°C. (**B**) CD spectra of divalent metal ion-mediated dimeric Chro-hairpin DNA duplex complexes. The drug concentration was 0.04 mM in a 20 mM Tris-HCl buffer (pH 7.3) at 20°C. The concentration of DNA was 0.04 mM. A synthetic hairpin DNA, TTGGCCAATGTTTGGCCAA, was used in the CD study (the hairpin loop is underlined).

The conformational changes of Chro upon binding to divalent metal ions were characterized using CD spectroscopy. As shown in **Figures 2A and 2B**, the CD spectra of Chro were scanned from 200 to 520 nm in the presence of various divalent metal ions, including Mg(II), Fe(II), Co(II), and Ni(II), without and with DNA binding. Based on the results of previous studies [30], [31], the spectral features in the 250–300 nm region most likely arose from the absorption assigned to the aglycon chromophore ring of Chro that was polarized along the long axis. Therefore, the intensity in the 250–300 nm region is indicative of a transition between the monomer and dimer of drugs in the aureolic family. In the absence of DNA binding, the CD spectra of the Chro monomer exhibited an obvious positive peak at 275 nm, and the inversion of the peak in the 250–300 nm region was observed during the formation of Fe^II^(Chro)_2_, Co^II^(Chro)_2_, and Ni^II^(Chro)_2_ in the presence of metal ions, with a concomitant decrease in the positive and negative peak intensities at 413 nm and 400 nm in the visible region, respectively (**Figure 2A**). The results presented here clearly indicate that a strong interaction exists between Chro and these three divalent metal ions because the electronic transition of the Chro chromophore was changed, as a result of the coordination of the divalent metal ions with the oxygen atoms of each chromophore ring of Chro. In addition, the change from monomer to dimer resulted in an inversion of the band at 290 nm attributed to Ni(II) and Co(II), which also differs from those arising from Fe(II), suggesting the conformations adopted by Co^II^(Chro)_2_ and Ni^II^(Chro)_2_ are significantly different from the conformation of Fe^II^(Chro)_2_. At the same concentration of Mg(II), Chro retains the monomer conformation. To monitor whether the integrity of the dimeric conformations of Fe^II^(Chro)_2_, Co^II^(Chro)_2_, and Ni^II^(Chro)_2_ are destroyed by high temperatures, the CD spectra of Fe^II^(Chro)_2_, Co^II^(Chro)_2_, and Ni^II^(Chro)_2_ at various temperatures (20, 40, and 60°C) were scanned from 520 to 200 nm, and the spectra are shown in **Figure S2**. No changes were observed in the CD spectra of Fe^II^(Chro)_2_ and Ni^II^(Chro)_2_ at these temperatures, suggesting that these two drug complexes remain stable in the dimeric conformation at high temperatures. Although the negative peak of Co^II^(Chro)_2_ at 290 nm was blue-shifted to 285 nm at 40 and 60°C under the same conditions, the intensity remained at the same level, suggesting that the dimer conformation of Co^II^(Chro)_2_ was largely preserved.

To characterize the effects of divalent metal ions on the conformation of the Chro dimer while interacting with DNA, dimeric Chro complexes were allowed to interact with DNA duplexes in the presence of various divalent metal ions, including Mg(II), Fe(II), Co(II), and Ni(II), and the interactions were monitored using CD spectroscopy (**Figure 2B**). The CD spectra of Fe^II^(Chro)_2_, Co^II^(Chro)_2_, and Ni^II^(Chro)_2_ bound to the DNA duplex exhibited similar spectral features, with negative and positive peaks at 275 and 287 nm, respectively, which provided good evidence for an octahedral coordination sphere around the metal ion [21]. The results also suggested that the dimer conformations of Chro are similar in the presence of various divalent metal ions during DNA binding.

### DNA affinity analyses of dimeric Chro complexes chelated with various divalent metal ions using SPR

**Figure 3:**
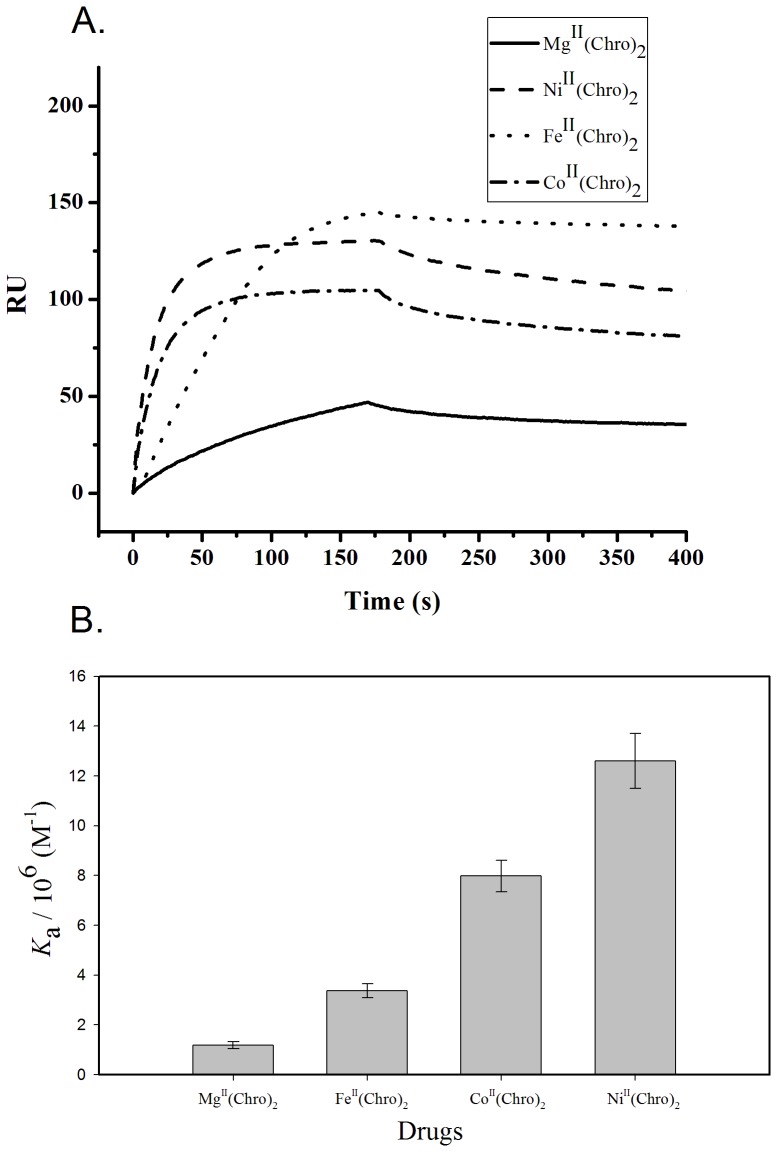
DNA affinity analyses of dimeric Chro complexes chelated with various divalent metal ions. (**A**) Sensorgrams of the interaction between an immobilized hairpin duplex and the target (5 µM) in a 20 mM Tris–HCl buffer (pH 7.3) containing 50 mM NaCl at 20°C. (**B**) Numerical values of the SPR-derived association equilibrium constants, *K*_a_, for immobilized hairpin DNA upon the binding of drugs.

To compare the binding affinity between DNA and the dimeric Chro complexes chelated with various divalent metal ions, the maximum binding capacity (*R_max_*) (in RU) and kinetic parameters were measured using SPR. To ensure the formation of the DNA duplexes in the SPR flow system, the biotin-labeled hairpin DNA duplexes providing one Chro DNA-binding site (GGCC) were used as the probe. According to the SPR sensorgram shown in **Figure 3A**, Fe^II^(Chro)_2_ appears to have a greater binding capacity (∼145 RU) than do the other dimeric Chro complexes, whereas the interaction between the hairpin DNA duplexes and Mg^II^(Chro)_2_ exhibits the lowest binding capacity (∼46 RU). The kinetic constants for the association (*k*_a_ in M^−1^s^−1^) and dissociation (*k*_d_ in s^−1^) of the dimeric Chro complex binding to the hairpin DNA duplexes were calculated from the association and dissociation phases of the SPR traces, respectively (**Table 1**). The Ni^II^(Chro)_2_ complex exhibited the highest *k*_a_, 1.11×10^4^ M^−1^ s^−1^, followed by Co^II^(Chro)_2_ and Fe^II^(Chro)_2_ with *k*_a_ values of 6.90×10^3^ and 2.63×10^3^ M^−1^ s^−1^, respectively. Mg^II^(Chro)_2_ exhibited the lowest *k*_a_ of 1.53×10^3^ M^−1^ s^−1^. As for the dissociation constants, the *k*_d_ values of Fe^II^(Chro)_2_, Co^II^(Chro)_2_, and Ni^II^(Chro)_2_ were essentially the same, with values of 7.80×10^−4^ s^−1^, 8.65×10^−4^ s^−1^, 8.78×10^−4^ s^−1^, respectively, although they were significantly smaller than the *k*_d_ value of Mg^II^(Chro)_2_, which was 1.30×10^−3^ s^−1^. The association constants (*K*_a_) of all of the dimeric Chro complexes binding to the hairpin DNA duplexes were calculated as *k*_a_/*k*_d_ (in M^−1^) and are graphed in **Figure 3B**. Ni^II^(Chro)_2_ exhibited the highest *K*_a_ with a value of 1.26×10^7^ M^−1^, approximately 1.6-fold, 3.7-fold, and 10.6-fold higher than the *K*_a_ values obtained for Co^II^(Chro)_2_, Fe^II^(Chro)_2_, and Mg^II^(Chro)_2_, respectively.

**Table 1:** Numerical values of the SPR-derived association rate constants, dissociation rate constants, and association equilibrium constants (*k*_a_, *k*_d_, and *K*_a_) for the immobilized hairpin DNA upon binding to a drug.

	*k*_a_(M^−1^s^−1^)	*k_d_* (s^−1^)	*K*_a_(M^−1^)
Mg^II^(Chro)_2_	1.53±0.12×10^3^	1.30±0.11×10^−3^	1.18±0.14×10^6^
Fe^II^(Chro)_2_	2.63±0.22×10^3^	7.80±0.28×10^−4^	3.37±0.29×10^6^
Co^II^(Chro)_2_	6.90±0.42×10^3^	8.65±0.51×10^−4^	7.98±0.63×10^6^
Ni^II^(Chro)_2_	1.11±0.09×10^4^	8.78±0.42×10^−4^	1.26±0.11×10^7^

### Stabilization effect of dimeric Chro chelated with divalent metal ions on the DNA duplexes

**Figure 4:**
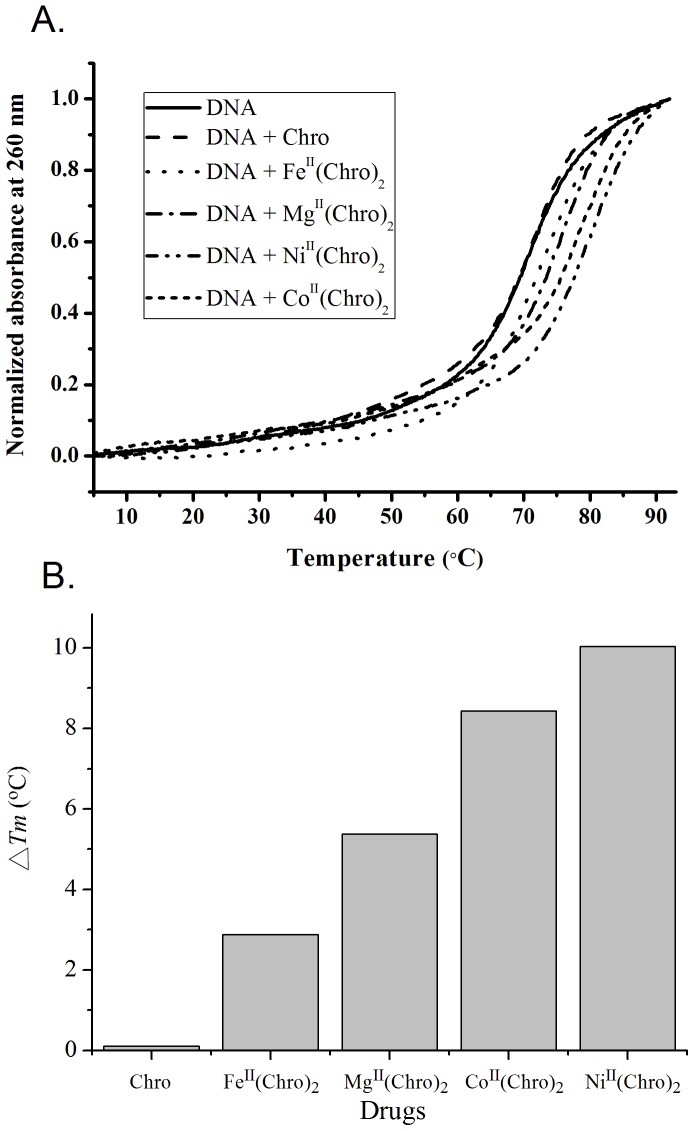
Stabilization effect of dimeric Chro chelated with divalent metal ions on the DNA duplexes. (**A**) The melting curves of the DNA duplexes (3 µM) with and without bound drugs in a 20 mM Tris-HCl buffer (pH 7.3) containing 50 mM NaCl at 20°C. (**B**) Comparison of melting temperature increases of hairpin DNA upon the binding of drugs.

To determine the stabilizing effects of dimeric Chro chelated by divalent metal ions on the formation thermodynamics of the DNA duplexes, the melting curves of the DNA duplexes were determined following their complexation by recording their A_260_ values at different temperatures (**Figure 4A**). The *T_m_* values of the DNA duplexes are almost identical in the absence and presence of Chro. The *T_m_* value of the duplex increased by 5.4°C upon the addition of Mg^II^(Chro)_2_ (**Figure 4B**). Although the binding affinity of Fe^II^(Chro)_2_ to DNA was greater than that of Mg^II^(Chro)_2_, the addition of Fe^II^(Chro)_2_ to the DNA duplex only resulted in an increase in the *T_m_* value of 2.9°C. Conversely, the addition of Co^II^(Chro)_2_ and Ni^II^(Chro)_2_ to the DNA duplex resulted in dramatic increases in the *T_m_* values of 8.4 and 10.0°C, respectively, because Co^II^(Chro)_2_ and Ni^II^(Chro)_2_ exhibit higher DNA-binding affinities compared to Mg^II^(Chro)_2_ and Fe^II^(Chro)_2_.

In addition, thermodynamic parameters, such as the Gibb's free energy change (Δ*G*), the enthalpy change (Δ*H*), the entropy change (Δ*S*), and the entropy change multiplied by absolute temperature (*T*Δ*S*), were derived from the thermal denaturation of the DNA duplex; the calculated values are listed in **Table 2**. The formation of the DNA duplex increased the entropy (Δ*S*) at 298 K in the presence of the dimeric Chro complexes. The enthalpy of DNA duplex formation is −292.1 kJ/mol. The enthalpies of DNA duplex formation in the presence of Co^II^(Chro)_2_ and Ni^II^(Chro)_2_ are −392 and −410.3 kJ/mol, respectively, which indicates that the DNA duplex is considerably more thermally stable upon Co^II^(Chro)_2_ and Ni^II^(Chro)_2_ binding. The extent of DNA duplex formation in the presence of the dimeric Chro complex is indicated by the Δ*G* value. The Δ*G* values for the formation of the DNA duplex examined here are negative, suggesting that the formation of DNA duplex in the presence and absence of drugs is an exergonic process. The Δ*G* value for the DNA duplex increased upon addition of the drug complexes in the following order: Ni^II^(Chro)_2_>Co^II^(Chro)_2_>Mg^II^(Chro)_2_>Fe^II^(Chro)_2_. Therefore, in terms of the melting of the DNA duplexes, the stabilizing effect of Ni^II^(Chro)_2_ on the DNA duplex is much greater than those of the other dimeric Chro complexes.

**Table 2:** The melting temperature and thermodynamic parameters derived from thermal denaturation for the formation of duplex DNA with and without drug binding at 25°C.

	*Tm* (°C)	Δ*G* (kJmol^−1^)	Δ*H* (kJmol^−1^)	−*T*Δ*S* (kJmol^−1^)
DNA alone	69.90 (±0.10)	−38.28 (±1.45)	−292.10 (±11.37)	253.82 (±9.94)
Chro	70.01 (±0.09)	−39.67 (±1.32)	−300.22 (±9.29)	260.55 (±8.22)
Fe^II^(Chro)_2_	72.79 (±0.05)	−43.47 (±0.63)	−314.00 (±4.70)	270.53 (±4.08)
Mg^II^(Chro)_2_	75.28 (±0.04)	−52.17 (±2.27)	−360.04 (±15.39)	307.86 (±13.11)
Co^II^(Chro)_2_	78.35 (±0.06)	−57.52 (±1.02)	−392.02 (±6.77)	334.48 (±5.73)
Ni^II^(Chro)_2_	79.94 (±0.05)	−63.57 (±1.60)	−410.34 (±10.01)	346.76 (±8.40)

### Plasmid DNA integrity assay in the presence of Fe(II)-, Co(II)-, and Ni(II)-containing dimeric Chro complexes

**Figure 5:**
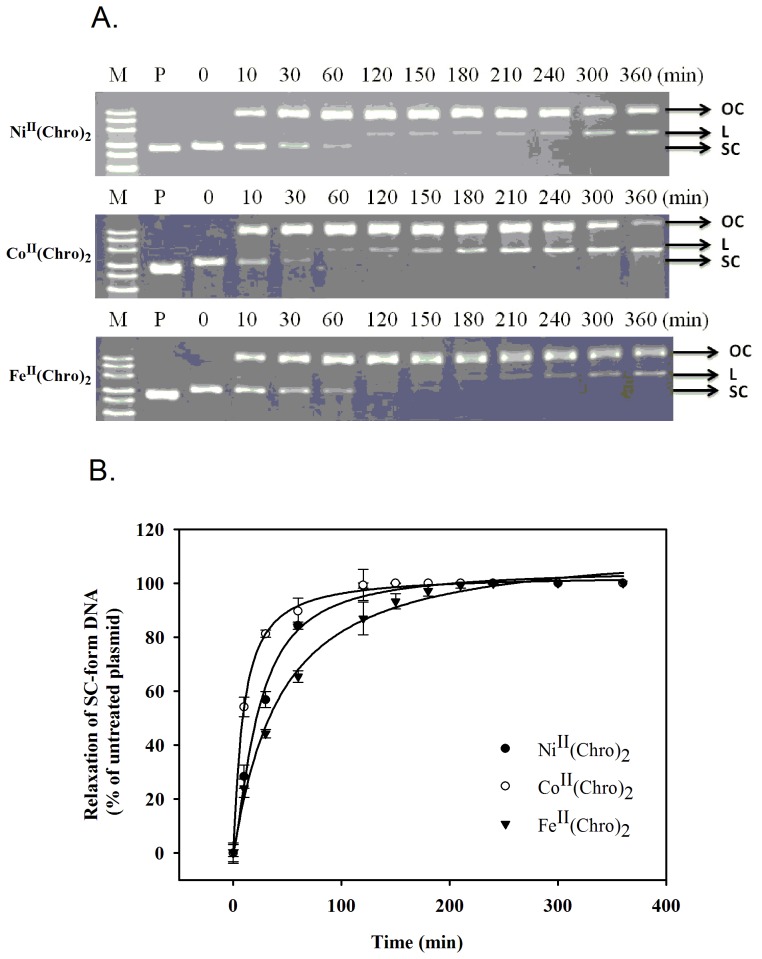
Plasmid DNA integrity assay in the presence of Fe(II)-, Co(II)-, and Ni(II)-containing dimeric Chro complexes. (**A**) Plasmid DNA (P) was treated with Co^II^(Chro)_2_, Fe^II^(Chro)_2_, or Ni^II^(Chro)_2_ (10 µM) and H_2_O_2_ at 37°C at various time points (0, 10, 30, 60, 120, 180, 240, 300 and 360 min). The supercoiled, open circular, and linear forms are denoted by SC, OC, and L, respectively. (**B**) Quantification of the percentage of plasmid cleavage relative to plasmid DNA per lane at various time points (0, 10, 30, 60, 120, 180, 240, 300 and 360 min). The cleavage rate constants (*k*_cl_) were calculated by fitting the normalized data to the equation [100- % cleavage] = 100e*^−kt^*, as described previously [51].

Previously, Co^II^(Chro)_2_ and Fe^II^(Chro)_2_ were shown to cause single-stranded cleavage of plasmid DNA [21]. The cleavage activity of Ni^II^(Chro)_2_ has remained unclear because the oxidation of nickel also results in the formation of reactive oxygen species, which can then cause oxidative damage to DNA [32]. Herein, to compare the DNA cleavage rate of the Fe(II)-, Co(II)-, and Ni(II)-containing dimeric Chro complexes, the plasmid DNA (P), pGEM-7zf(−), was treated with these complexes (10 µM) in the presence of H_2_O_2_ at various time points by monitoring the levels of conversion of the DNA from the supercoiled (SC) form to the open circular (OC) and linear (L) forms, as shown in **Figure 5A**. Untreated plasmid was observed as a single supercoiled (SC) DNA band on gels. No plasmid DNA relaxation by the Fe(II)-, Co(II)-, and Ni(II)-containing dimeric Chro complexes was observed in the absence of H_2_O_2_, suggesting that the DNA cleavage activities of these drug complexes was achieved via a Fenton-type reaction. The SC form of plasmid DNA was completely converted into the OC and L forms by Ni^II^(Chro)_2_, Co^II^(Chro)_2_, and Fe^II^(Chro)_2_ at 120, 60, and 150 min, respectively. Moreover, the levels of the L form of plasmid DNA caused by Ni^II^(Chro)_2_, Co^II^(Chro)_2_, and Fe^II^(Chro)_2_ increased with time. These results confirmed the observation that plasmid breakage occurs as a result of the formation of double-strand scissions, which result from the stepwise cleavage of single strands. The DNA cleavage rate of Co^II^(Chro)_2_ (1.2×10^−3^ s^−1^) is higher than those of Fe^II^(Chro)_2_ and Ni^II^(Chro)_2_, which were calculated to be 1×10^−4^ and 3.1×10^−4^ s^−1^, respectively (**Figure 5B**).

### Inhibition of in vitro transcription by Fe(II)-, Co(II)-, and Ni(II)-containing dimeric Chro complexes

**Figure 6:**
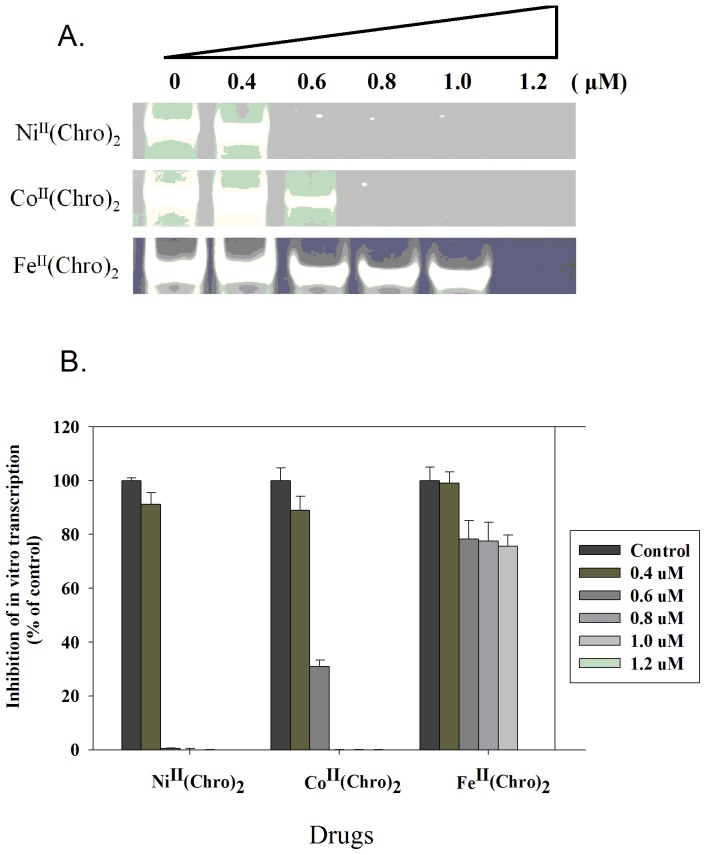
Inhibition of in vitro transcription by Fe(II)-, Co(II)-, and Ni(II)-containing dimeric Chro complexes. (**A**) The effects of Co^II^(Chro)_2_, Fe^II^(Chro)_2_, and Ni^II^(Chro)_2_ at various concentrations on T7 RNA polymerase activity. (**B**) Quantification of the percentage of RNA polymerase activity on drug inhibition at various concentrations relative to the control (no drug treatment). The data represent the mean values ±SDs from three separate experiments.

To compare the inhibitory effects of Ni^II^(Chro)_2_, Co^II^(Chro)_2_, and Fe^II^(Chro)_2_ on *in vitro* transcription, pTRI-β-actin-mouse cDNA was used as a template and was treated with T7 RNA polymerase to monitor transcription in the presence of increasing concentrations of the drug complexes. T7 polymerase is a good model for *in vitro* transcription due to its extreme promoter specificity [33]. In the absence of drug complexes, a 245-bp β-actin mRNA product was produced by T7 RNA polymerase (**Figure 6A**). In the presence of increasing concentrations of Ni^II^(Chro)_2_, Co^II^(Chro)_2_, and Fe^II^(Chro)_2_, the level of the RNA product was diminished (**Figure 6B**). The synthesis of RNA molecules by the T7 RNA polymerase was completely inhibited by Ni^II^(Chro)_2_ and Co^II^(Chro)_2_ at a concentration of 0.6 and 0.8 µM, respectively (**Figure 6A**). The enzymes displayed approximately 68% inhibition by Co^II^(Chro)_2_ at concentrations of 0.6 µM. In addition, T7 RNA polymerase was completely inhibited by 1.2 µM Fe^II^(Chro)_2_ (**Figure 6A**).

### Comparison of c-myc transcription inhibition by Fe(II)-, Co(II)-, and Ni(II)-containing dimeric Chro complexes in HeLa cancer cells

**Figure 7:**
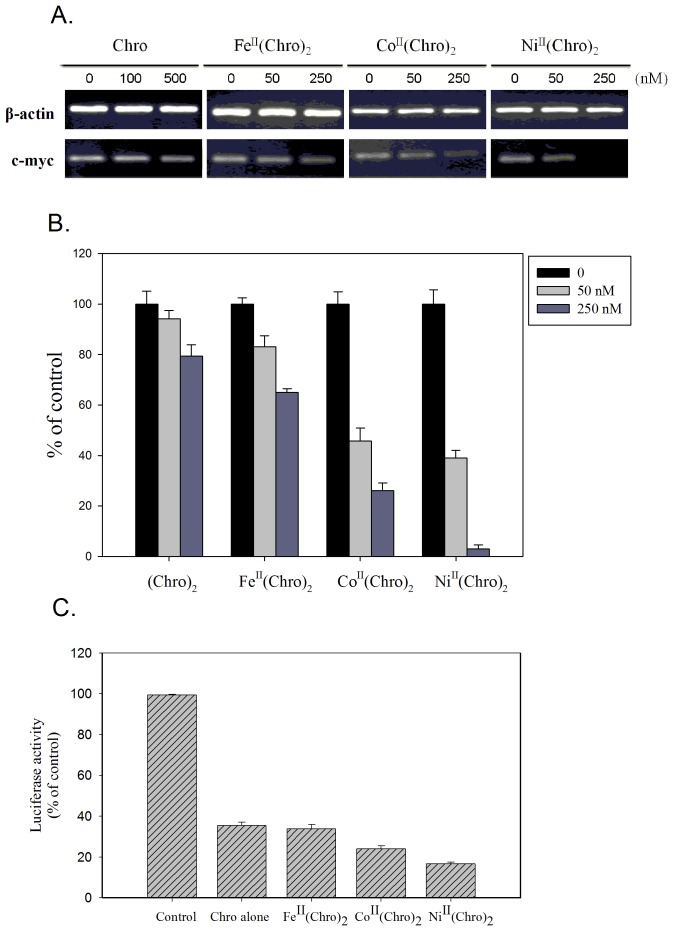
c-myc transcription inhibition by Fe(II)-, Co(II)-, and Ni(II)-containing dimeric Chro complexes in HeLa cancer cells. (**A**) The effects of Chro alone (100 and 500 nM), Co^II^(Chro)_2_ (50 and 250 nM), Fe^II^(Chro)_2_ (50 and 250 nM), and Ni^II^(Chro)_2_ (50 and 250 nM) on the c-myc transcripts of HeLa cells determined using RT-PCR for 24 h. β-actin was the internal control. (**B**) Quantification of the percentage of c-myc transcripts during drug inhibition at various concentrations relative to the control sample (no drug treatment). The data represent the mean values ±SDs from three separate experiments. (*p<0.05). (**C**) Normalized luciferase activity observed in HeLa cells transfected with the c-myc promoter luciferase reporter vector after treatment with Chro alone (0.2 µM), Co^II^(Chro)_2_ (0.1 µM), Fe^II^(Chro)_2_ (0.1 µM), and Ni^II^(Chro)_2_ (0.1 µM) at 18 h.

To compare the transcription inhibition by Ni^II^(Chro)_2_, Co^II^(Chro)_2_, and Fe^II^(Chro)_2_ in a cell model, we monitored c-myc gene expression in HeLa cells using RT-PCR in the presence of increasing concentrations of drug complexes because Chro has been reported to inhibit the transcription of c-myc by binding to a GC element in the c-myc promoter, primarily inhibiting the Sp1 family from binding to DNA (**Figure 7A**) [34]. Uncomplexed Chro was used as a control. Free Chro at 100 and 500 nM inhibited transcription of the c-myc gene by 6 and 21%, respectively (**Figure 7B**). Cells cultured in the presence of 50 and 250 nM Fe^II^(Chro)_2_ for 6 h displayed ∼17 and 26% reductions in c-myc expression, respectively, whereas the addition of 50 and 250 nM Co^II^(Chro)_2_ reduced c-myc levels by ∼55 and 74% (**Figure 7B**). These results indicate that Co^II^(Chro)_2_ and Fe^II^(Chro)_2_ are both able to inhibit c-myc expression in HeLa cells. Moreover, Ni^II^(Chro)_2_-treated HeLa cells markedly reduced c-myc gene expression by 61 and 98% in the presence of 50 and 250 nM Ni^II^(Chro)_2_, respectively (**Figure 7B**). These results suggest that Ni(II), Co(II), and Fe(II) potentiate the inhibitory activity of Chro on the expression of the c-myc gene in the cancer cells. Ni^II^(Chro)_2_ inhibited transcription at lower concentrations and to a higher degree than did Co^II^(Chro)_2_ and Fe^II^(Chro)_2_.

To confirm the inhibition by Ni^II^(Chro)_2_, Co^II^(Chro)_2_ and Fe^II^(Chro)_2_ on c-myc expression via binding of the metal complex to the c-myc promoter region, we used a reporter assay using a human c-myc promoter-driven luciferase system to compare the capacities of the metal derivatives of dimeric Chro to block the transcription driven by GC-rich DNA-binding transcription factors (**Figure 7C**). Ni^II^(Chro)_2_ was the most potent compound in the reporter assays, inducing >80% inhibition at a concentration of 100 nM. At this dose, Co^II^(Chro)_2_ and Fe^II^(Chro)_2_ inhibited the c-myc promoter-based reporter by only 75 and 65%, respectively.

### Comparison of the cytotoxicity of the Fe(II)-, Co(II)-, and Ni(II)-containing dimeric Chro complexes in the HepG2 cancer cell line

To evaluate the antitumor effects of the metal complexes examined in this study, cell viability was assessed in HeLa, HSC-3, and A549 cells using an MTS assay involving the three metal-derived dimeric Chro complexes; uncomplexed Chro was used as a control. Treatment of three cell lines for 24 h with 10 µM FeSO_4_, CoCl_2_, and NiSO_4_ alone did not visibly affect the cell viability. **Table 3** shows the IC_50_ values of the HeLa, HSC-3, and A549 cells after incubation with the three metal-derived dimeric Chro complexes for 24 h. The HeLa cells appeared to be more sensitive to these drug complexes relative to other cancer cells. Fe(II), Co(II), and Ni(II) potentiated the antiproliferation effects of Chro in HeLa cells at 24 h. These values showed more than two-fold cytotoxicity compared to those of Chro treatment alone at 24 h. Moreover, Ni^II^(Chro)_2_ was shown to be more active toward HeLa cells, with an IC_50_ value of 52.5±6.6 nM at 24 h, than were Co^II^(Chro)_2_ and Fe^II^(Chro)_2_, with IC_50_ values of 90.0±2.5 nM and 208.7±3.8 nM, respectively (**Table 3**). In addition, we tested the cytotoxicity of the three metal-derived dimeric Chro complexes toward other cancer cell lines, including HSC-3 and A549 cells, under the same conditions as described for HeLa cells (**Table 3**). As expected, Ni^II^(Chro)_2_ had better anti-proliferation effects on HSC-3 and A549 cells than did Co^II^(Chro)_2_ or Fe^II^(Chro)_2_, with IC_50_ values of 78.0±1.9 nM and 413.5±4.5 nM, respectively, at 24 h.

**Table 3:** Effects of Chro and the Ni^II^-, Co^II^-, Fe^II^-containing dimeric Chro complexes on the viability of various cancer cell lines at 24 h; reported as IC_50_ a values (nM).

	HeLa	HSC-3	A549
Chro	468.6±11	709.2±30.6	1324.8±35.8
Fe^II^(Chro)_2_	208.7±3.8	333.9±6.1	676.6±6.1
Co^II^(Chro)_2_	90.0±2.5	187.1±9.1	615.0±16.1
Ni^II^(Chro)_2_	52.5±6.6	78.0±1.9	413.5±4.5

a IC_50_ indicates the concentrations that inhibited cell growth by 50%.

## Discussion

Recently, the use of the aureolic family of drugs has emerged in both cancer- and non-cancer-related disease therapies [35], [36]. For example, the new aureolic acid-type compounds, generated via combinatorial biosynthetic methods, were investigated for their application in cancer therapy and their effect on Sp binding to the c-src promoter region [37]. The combination of betulinic acid and mithramycin results in a synergistic inhibitory effect on pancreatic cancer growth and has a therapeutic advantage over gemcitabine [38], [39]. In addition, Lahiri et al. indicated that Chro has potential for chelation therapy in Cu(II) accumulation [40]. Divalent metal ions have been shown to be necessary for dimer formation of drugs of the aureolic family prior to the binding of these drugs to a GC-rich DNA duplex [11], [22]. Recently, we focused on the development of metal-derived complexes of the aureolic family of drugs and the exploration of their multifunctional anticancer activities, including DNA cleavage activity and topoisomerase I inhibition [4], [21], [24], [41]. In this study, the dimer form of Chro chelated with Fe(II), Co(II), and Ni(II) can be achieved when Fe(II), Co(II), and Ni(II) are in low-spin states with ionic radii of 75, 79, and 83 pm, respectively [20]. It is believed that the rigid steric configuration of the bidentate Chro complex containing two neutral oxygen atoms on the chromophore causes a large ligand field splitting on the central metal ions and results in the low-spin states of the chelated divalent metal ions. In stark contrast to the formation of Fe(II)-containing dimeric Chro complexes that exhibited an inversion of the CD band at approximately 275 nm during complex formation, the formation of the Co(II)- and Ni(II)-containing dimeric Chro complexes caused the negative peak at 290 nm. The divalent metal ion-dependent difference in the CD spectra of Chro was ascribed to different structures of the dimeric Chro complexes chelated with various divalent metal ions, specifically the coordination geometry. When metalloantibiotics are used in a clinical trial, their structural integrity must be maintained. Recently, our CD spectral analyses showed that the more stable dimeric complexes of Chro chelated with Fe(II), Co(II), and Ni(II) exhibited strong structural integrity against destruction by heat at 60°C.

Previous studies have shown that metal ions play a crucial role in determining the DNA reactivity of several synthetic and natural metalloantibiotics [10]. To explore the effects of various divalent metal ions, including Fe(II), Co(II), and Ni(II), on the efficacy of dimeric Chro complexes in the nucleus of cancer cells, we further characterized the DNA reactivity of these complexes. According to SPR assays, the analysis of the kinetic parameters reveals marked differences among Co^II^(Chro)_2_, Fe^II^(Chro)_2_, and Ni^II^(Chro)_2_ interacting with a DNA duplex containing regions of 4-bp G-tracts. Ni^II^(Chro)_2_ exhibits a faster association rate for binding to DNA than do the other complexes, whereas the dissociation rates of Co^II^(Chro)_2_, Fe^II^(Chro)_2_, and Ni^II^(Chro)_2_ from the DNA were essentially the same. This result suggests that a suitable minor groove in the DNA duplex allows easier access to the minor groove for Ni^II^(Chro)_2_ than for other complexes. Moreover, the binding affinity of the dimeric Chro complexes for the DNA duplex follows the order Ni^II^(Chro)_2_>Co^II^(Chro)_2_>Fe^II^(Chro)_2_. The thermal stability of the dimeric-Chro-M DNA duplexes increases considerably (Δ*T*_m_ = 2.89–10.04°C) depending on the metal ion compared to the stability of the complexes without metal ions. Consistent with the above SPR results, the most dramatic increases in the *Tm*, Δ*G* and Δ*H* values of the DNA duplex were observed upon Ni^II^(Chro)_2_ binding relative to the other complexes. Moreover, this study provides evidence that Co^II^(Chro)_2_, Fe^II^(Chro)_2_, and Ni^II^(Chro)_2_ can generate damaging hydroxyl radicals that cause DNA stand breaks via a Fenton-type reaction and can kill cancer cells. Interestingly, the Fe^II^(Chro)_2_-induced DNA cleavage rate was 12 and 3.1 times lower than those of Co^II^(Chro)_2_ and Ni^II^(Chro)_2_, respectively. Although Co^II^(Chro)_2_, Fe^II^(Chro)_2_, and Ni^II^(Chro)_2_ exhibited different DNA reactivities, the CD spectra of Fe(II)-, Co(II)-, and Ni(II)-containing dimeric Chro complexes bound to DNA were essentially identical to that of a Mg^II^(Chro)_2_ complex in the presence of a DNA duplex, exhibiting induced CD intensities at 287 and 275 nm (UV region). The results also suggest that these complexes have similar binding modes toward DNA.

RNA polymerase, which is necessary for constructing RNA chains using DNA genes as templates in essential cellular processes, is the target of many therapeutic agents [42]. Various RNA polymerase inhibitors, such as actinomycin D, are presently used as drugs in cancer therapy [43]. T7 RNA polymerase possesses all of the fundamental features of eukaryotic RNA polymerases and serves as an ideal model system in which to study the functional mechanisms of transcription *in vitro* [44], [45]. Our study showed that Co^II^(Chro)_2_, Fe^II^(Chro)_2_, and Ni^II^(Chro)_2_ were able to interfere with the activity of T7 RNA polymerase via groove binding. The binding of a Chro-metal complex with DNA could prevent RNA and DNA polymerase from binding to the DNA, thus affecting the initiation of transcription. In addition, the drug can block the progression of RNA polymerase along the template DNA, prematurely terminating transcription. In this study, Ni^II^(Chro)_2_ possesses the highest inhibitory effect on RNA polymerase *in vitro* compared to the other complexes because it exhibits the highest DNA-binding affinity.

Transcription factors, such as Sp1 bound to GC-rich sequences in promoter regions, are associated with cancer pathogenesis [46]. Blocking the binding of transcription factors to DNA using DNA-binding drugs and, in turn, modulating the expression of oncogenes such as c-myc have become attractive issues in cancer therapy [47]. Previous studies reported that the aureolic acid antibiotic Mith with GC-rich DNA sequences selectively inhibits c-myc gene transcription by interacting with the c-myc promoter to block the access of the Sp1 transcription factor [48]. Our current RT-PCR and luciferase reporter studies have shown that Co^II^(Chro)_2_, Fe^II^(Chro)_2_, and Ni^II^(Chro)_2_ have the capacity to inhibit c-myc gene transcription regulated by the Sp1 transcription factor. As a consequence of our *in vitro* transcription results, which showed the highest inhibitory effect on the RNA polymerase exhibited by Ni^II^(Chro)_2_, this Ni-containing drug complex has the pronounced capacity to inhibit c-myc transcription via binding to the c-myc promoter region.

In early studies, complexes of the structurally related DNA-binding anticancer antibiotics with metals have been tested to amplify their potential in cancer therapy [49], [50]. For example, doxorubicin, one of the best known anticancer drugs, has been tested regarding its mode of complexation with a number of metal ions, such as Pt(II), Fe(III) and Cu(II), to determine its effects on tumor cells [10]. Here, we found that changes in the divalent metal ions in the dimeric Chro complexes have been correlated with improved anticancer profiles. By comparing the cytotoxicities of Co^II^(Chro)_2_, Fe^II^(Chro)_2_, and Ni^II^(Chro)_2_ toward several cancer cell lines, our studies concluded that Chro in the presence of Ni(II) exhibited more potential antitumor activities than did Co^II^(Chro)_2_ and Fe^II^(Chro)_2_, due to its higher DNA-acting efficacy. The availability of new metal derivatives of Chro with improved pharmacological profiles may introduce new possibilities for exploiting the unique properties of this class of compounds for therapeutic applications. In summary, our results may provide important insights into the properties of stable dimeric complexes containing Chro chelated by divalent metal ions that may be applied to cancer therapy in the future.

## Supporting Information

Figure S1 **The schematic structure of divalent metal ion (M^2+^)-coordinated dimeric complex of Chro.** Divalent metal ion (M^2+^) is octahedrally coordinated to two oxygen atoms of both chromophore, while two water molecules act as fifth and sixth ligands.
(TIF)

Figure S2 **The integrity of the dimeric conformations of Fe^II^(Chro)_2_, Co^II^(Chro)_2_, and Ni^II^(Chro)_2_ at various temperatures.** The CD spectra of (**A**) Fe^II^(Chro)_2_, (**B**) Co^II^(Chro)_2_, and (**C**) Ni^II^(Chro)_2_ in a 20 mM Tris-HCl buffer (pH 7.3) at 20, 40, 60°C.
(TIF)
